# *Dendrobium officinale* Polysaccharide Protected CCl_4_-Induced Liver Fibrosis Through Intestinal Homeostasis and the LPS-TLR4-NF-κB Signaling Pathway

**DOI:** 10.3389/fphar.2020.00240

**Published:** 2020-03-12

**Authors:** Kaiping Wang, Xiawen Yang, Zhijing Wu, Hongjing Wang, Qiang Li, Hao Mei, Ruxu You, Yu Zhang

**Affiliations:** ^1^Hubei Key Laboratory of Natural Medicinal Chemistry and Resource Evaluation, Tongji Medical College of Huazhong University of Science and Technology, Wuhan, China; ^2^Department of Pharmacy, Union Hospital of Huazhong University of Science and Technology, Wuhan, China; ^3^Puai Hospital, Tongji Medical College of Pharmacy, Huazhong University of Science and Technology, Wuhan, China

**Keywords:** *Dendrobium officinale* polysaccharide, liver fibrosis, intestinal tight junction, apoptosis, TLR4/NF-κB pathway

## Abstract

We explored the therapeutic effects of *Dendrobium officinale* polysaccharide (DOP) on CCl_4_-induced liver fibrosis with respect to the intestinal hepatic axis using a rat model. Histopathological staining results showed that DOP alleviated extensive fibrous tissue proliferation in interstitium and lessened intestinal mucosal damage. Western blot and PCR results showed that DOP maintained intestinal balance by upregulating the expression of tight junction proteins such as occludin, claudin-1, ZO-1, and Bcl-2 proteins while downregulating the expression of Bax and caspase-3 proteins in the intestine. The transepithelial electrical resistance (TEER) value of the LPS-induced Caco-2 monolayer cell model was increased after DOP administration. These illustrated that DOP can protect the intestinal mucosal barrier function. DOP also inhibited activation of the LPS-TLR4-NF-κB signaling pathway to reduce the contents of inflammatory factors TGF-β and TNF-α, increased the expression of anti-inflammatory factor IL-10, and significantly decreased α-SMA and collagen I expression. These results indicated that DOP maintained intestinal homeostasis by enhancing tight junctions between intestinal cells and reducing apoptosis, thereby inhibiting activation of the LPS-TLR4-NF-κB signaling pathway to protect against liver fibrosis.

## Introduction

Liver fibrosis is a common disease related to viral hepatitis, alcoholic liver, autoimmune disease, metabolic disease and cholestasis liver disease, that often leads to the gradual loss of liver function (Friedman, 2003; Shiha et al., 2017). Liver fibrosis is due to the excessive deposition of extracellular matrix (ECM), mainly from activated hepatic stellate cells (HSCs) (Gan et al., 2018). Collagen I is the most abundant extracellular matrix component in fibrosis (Schuppan, 2015). HSC cells are the most activated cells in liver fibrosis and involve in collagen synthesis, and alpha-smooth muscle actin (α-SMA) is the sign of HSC cell activation (Chiu et al., 2014). Many small molecules are important triggers of liver fibrosis, such as tumor necrosis factor-alpha (TNF-α), interleukin-10 (IL-10), and transforming growth factor-beta (TGF-β), which possess effective pro-inflammatory or anti-inflammatory effects (Seki and Brenner, 2015; Kim et al., 2016; Al-Hashem et al., 2019; Xu et al., 2019). Inflammation promotes the activation of HSC cells and aggravates liver fibrosis, ultimately leading to cirrhosis of the liver, which endangers human life (Mitra et al., 2014). Therefore, the prevention and treatment of liver fibrosis are important steps in liver cirrhosis clinical therapy.

The liver is the first parenteral organ that the portal vein encounters when it collects blood flow from the veins of the small intestine and the large intestine. This unique blood supply system makes the liver very receptive to antigen stimulation from the intestinal cavity (Doherty, 2016). A mechanical barrier composed of intestinal epithelial cells and tight junctions (TJs) is the structural basis and core content of the intestinal mucosal barrier (Odenwald and Turner, 2013). Once intestinal TJs are destroyed, the intestinal mucosal permeability will increase, which elevates LPS contents in the liver (Seki and Schnabl, 2012). Toll-like receptor 4 (TLR4) is a congenital immune receptor expressed in hepatocytes. When LPS enters the liver, it synergizes with TLR4 and ultimately causes nuclear factor-κB (NF-κB) translocation, which is a highly conserved and tightly regulated signaling pathway central to liver survival and homeostasis that regulates the expression of inflammatory factors and induces liver inflammation (Porras et al., 2017; Zusso et al., 2019). Overall, the LPS-TLR4-NF-κB pathway is not only a bridge between the intestinal homeostasis and liver inflammation, but also provides the possibility to reduce liver diseases by protecting intestinal function (Chassaing et al., 2014).

As previously described, liver disease is the leading cause of illness and death all over the world (Wang et al., 2014). Functional food or its natural bioactive components have become a relatively good choice for chemotherapeutic patients because of the side-effects of pharmaceutical products; for example, as a functional food that promotes good health and longer life, *Pleurotus eryngii* var. *tuoliensis*, has hepatoprotective effects and antioxidant activity (Xu et al., 2017). Moreover, polysaccharide from *Ganoderma lucidum* ameliorate cognitive function (Huang et al., 2017). The liver is in closest contact with the intestinal tract (Wood, 2014). Natural polysaccharides can promote the development of epithelial cells and are a class of nutrients that can affect the function of the intestinal mucosal barrier (Xie et al., 2019). Polysaccharides can promote the growth of probiotics, and can also be used as prebiotics in daily diet, which can promote intestinal biodiversity (Zhang et al., 2018). *Dendrobium officinale* is an important multifunctional food in China and has health promoting functions (Xing et al., 2015). The pharmacological effects of *Dendrobium officinale* polysaccharide (DOP) are mainly manifested as immunity, hypoglycemic effect, anti-cancer, anti-oxidation, anti-inflammation, anti-senility and so on, with broad application prospects (He et al., 2016; Wang et al., 2018; Wang H.Y. et al., 2019; Liang et al., 2019). Previous research have indicated that DOP could protect intestinal mucosal barrier function and fight against acetaminophen-induced liver injury (Xing et al., 2015; Lin et al., 2018). Additionally, DOP has a wide range of sources, a clear structure, and a green and safe extraction process. Thus, we wanted to explore the influence of DOP on the gut-liver axis.

The purpose of this study was to explore the effects of DOP on hepatic fibrosis in a CCl_4_-induced hepatic fibrosis rat model and an LPS-induced Caco-2 epithelial cell model. The effects of DOP on intestinal mucosal barrier function were studied to further demonstrate its protection against rat hepatic fibrosis and provide a new potential mechanism. Furthermore, we detected the levels of TLR4, NF-κB, TNF-α, TGF-β, IL-10, α-SMA, and collagen I to study the degree of liver fibrosis in rats. Those findings demonstrated that DOP could be an alternative functional food to ameliorate hepatic fibrosis.

## Materials and Methods

### Materials

*Dendrobium officinale* Kimura et Migo (Orchidaceae) was purchased from Yueqing (Zhejiang Province, China) and the specimen (TJ-DO-20180330) was conserved in the Herbarium, No. 144, Tongji Medical College, Huazhong University of Science and Technology (HUST) (Wuhan, China). The Institute of Biochemistry and Cell Biology provided us with Caco-2 cell line (Shanghai, China), The aspartate aminotransferase (AST), alanine aminotransferase (ALT), lactate dehydrogenase (LDH), alkaline phosphatase (AKP) kits were obtained from Jiancheng Bioengineering Institute (Nanjing, China). An LPS kit was purchased from Xiamen Bioendo Technology Co., Ltd. (Xiamen Province, China). The primary antibodies, including anti-ZO-1 (ab61357), anti-occludin (ab167161), anti-claudin-1 (ab180158), anti-Bax (CST#2772T), anti-Bcl-2 (12789-1-AP), anti-caspase-3 (CST#9661T), anti-NF-κB (CST#8242T), anti-IκBα (ab32518), anti-p-IκBα (CST#2859T), anti-α-SMA (CST#19245S), anti-collagen I (ab34710), anti-β-actin (ab8227), anti-Histone H3 (CST#9728) and anti-GAPDH (ab8245) were obtained from Cell Signaling Technology (Danvers, MA, United States) or Abcam (Cambridge, England). The anti-rabbit IgG (CST#7074S) or anti-mouse IgG (CST#7076S) secondary antibody were purchased from Cell Signaling Technology. The nuclear-cytosol extraction kit was obtained from Thermo Scientific (Rockford, IL, United States).

### Preparation and Purification of DOP

The polysaccharide was extracted from the dry roots of *Dendrobium officinale*, and the dry roots were broken into pieces. The rude polysaccharide was extracted by water extraction and ethanol precipitation, After repeated freeze-thawing, ultrafiltration and freeze-drying, purified DOP was obtained (Wang et al., 2018). Plant was identified at Tongji Medical College of HUST, Wuhan, China.

### Structural Characterization of DOP

We used the phenol-sulfuric acid method to determine the sugar content. The molecular weight of DOP was determined by HPGPC (Agilent-LC, Agilent, United States). Nucleic acids and proteins were detected in the 200–400 nm range using a UV spectrophotometer. The composition of monosaccharide was determined using a modified PMP labeling method (Zhang et al., 2019). The detailed method is shown in Supplementary Materials.

### Establishment of the CCl_4_-Induced Liver Fibrosis Model

Male Sprague-Dawley (SD) rats (200 ± 20 g) were acquired from the Animal Center of Disease Control and Prevention (Wuhan, China) [Reg. No. SCXK (Hubei) 2016-0009]. All procedures were performed in accordance with the approval and guidelines of the Animal Research Committee of Tongji Medical College.

The experiment followed a previously reported model and is described as follows (Fang et al., 2008). After 1 week of free diet, we divided rats into the following five groups (*n* = 12): (1) Normal control group (NC), rats were treated with 2 mL/kg 5% peanut oil (two times/week); (2) Fibrotic control group (FC), rats were treated with 2 mL/kg 30% CCl_4_ (two times/week); (3) Low DOP treated group (LDOP), rats were treated with 2 mL/kg 30% (two times/week) CCl_4_ and 2 mL 200 mg/kg DOP solution; (4) Middle DOP treated group (MDOP), rats were treated with 2 mL/kg 30% CCl_4_ (two times/week) and 2 mL 400 mg/kg DOP solution; and (5) High DOP treated group (HDOP), rats were treated with 2 mL/kg 30% CCl_4_ (two times/week) and 2 mL 800 mg/kg DOP solution. The FC group was given an intraperitoneal injection of CCl_4_ for 6 weeks and free diet for 2 weeks, and the treatment groups were given different doses of DOP by oral administration for 8 weeks. The DOP was dissolved in distilled water. During the experiment, all rats were allowed to eat the normal diet freely and kept under ambient temperature conditions and light conditions.

### Determination of Biological Indicators in Serum and HYP Contents in Liver Homogenate

The AST, ALT, LPS levels in serum and HYP content in liver homogenates were detected using the kits according to the manufacturers’ instructions.

### Histological Analysis and Immunohistochemistry

The liver and intestinal tissues were removed, fixed in 10% formalin and embedded in paraffin wax. Paraffin sections with thickness of 5 μm were prepared, the liver tissue sections were stained with hematoxylin-eosin (H&E) and Masson’s trichrome staining methods (Huang et al., 2019). Histological and pathological changes were studied using a light microscope.

For immunohistochemistry, the paraffin-embedded liver and intestinal tissues (4 μm sections) were first incubated overnight with anti-claudin-1, anti-α-SMA, and anti-collagen I antibodies. And then, the sections were incubated for 60 min with horseradish peroxidase (HRP)-conjugated secondary antibody, stained with diaminobenzidine (DAB) as peroxidase substrate for 3–15 min, and restained with hematoxylin (Fan et al., 2018).

### Quantitative Real-Time PCR

Total RNA from liver was isolated using the Trizol reagent (Invitrogen, United States) and the RNA concentration was measured with a NanoDrop (Thermo Fisher Scientific, United States). The first-strand cDNA was synthesized using Prime Script RT Master Mix (Takara, RRO36A), and SYBR Green (BioEasy Master Mix, BIOER) was used to perform real-time PCR in a Real-time PCR System (BioRad) (Yuan et al., 2017). The following are the primer sequences for real-time PCR:

TNF-α-F, 5′-ATGAGCACAGAAAGCATGATC-3′;
TNF-α-R,5′-TACAGGCTTGTCACTCGAATT-3′;
TGF-β1-F, 5′-AGGGCTACCATGCCAACTTC-3′;
TGF-β1-R, 5′-CCACGTAGTAGACGATGGGC-3′;
collagen-I-F, 5′-GACGCCATCAAGGTCTACTG-3′;
collagen-I-R, 5′-ACGGGAATCCATCGGTCA-3′;
TLR4-F, 5′-ATGGCATGGCTTACACCACC-3′;
TLR4-R, 5′-GAGGCCAATTTTGTCTCCACA-3′;
IL-10-F, 5′-CTTACTGACTGGCATGAGGATCA-3′;
IL-10-R, 5′-GCAGCTCTAGGAGCATGTGG-3′;
β-actin-F, 5′-GACGGCCAGGTCATCACTATTG-3′;
β-actin-R, 5′-CCACAGGATTCCATACCCAAGA-3′.
The results were normalized to the house-keeping gene β-actin.

### Cell Culture

Caco-2 cells were cultured in Dulbecco’s modified Eagle’s medium (DMEM) supplemented with 10% fetal bovine serum (FBS), 1% penicillin-streptomycin, and 1% non-essential amino acids at 37°C in 5% CO_2_ atmosphere (Yang et al., 2017). Cells were cultured on appropriate plates to measure the transepithelial electrical resistance (TEER) and permeability or for testing by western blot or with an apoptotic detection kit.

### Measurement of Monolayer Transepithelial Electrical Resistance (TEER)

Cell transmembrane resistance was measured using a Millicell ERS-2 (Millipore, Sigma) (Wang J. et al., 2019). Caco-2 cells were seeded onto Transwell plates, and D-hanks was used as a control group with non-cell. The TEER was measured at days 0, 7, 10, 13, 15, 17, and 21. When the monolayer cells were completely differentiated, the cells were cocultured with 2 μg/mL LPS and 0, 50, 100, 200 μg/mL DOP simultaneously, referred to as the LPS, LDOP, MDOP, and HDOP groups, respectively. The TEER was measured at 0, 2, 4, 7, and 10 h after treatment. TEER value = (Experimental hole−blank hole) × 1.12 cm^2^. The data were set to 100% with 0 h as the reference value, and the other data were the percentage of the decrease or increase.

### Determination of AKP Contents and LDH Release Assay

An AKP assay kit was used to detect the enzyme activity of apical (AP) side and basolateral (BL) side in the Transwell plates at days 7, 13, and 17. Before and after LPS and DOP intervention, an LDH assay kit was applied to measure the LDH leakage in Transwell chambers to observe the degree of cell membrane damage.

### Preparation of Total Protein, Cytoplasm, and Nuclear Extracts

Caco-2 cells were planted into 6-well plates for 24 h and treated with 2 μg/mL LPS together with different concentrations of DOP (0, 50, 100, and 200 μg/mL) for 2 days. RIPA buffer (Beyotime Technology, P0013B) was added to the total cells, liver or intestinal tissues at a ratio of 0.1 g/mL with 1% protease inhibitors, phosphatase inhibitors and PMSF (Cali et al., 2018). We used the BCA concentration kit to quantify protein concentration (Beyotime Technology, P0010S). Cytoplasmic and nuclear extracts were isolated from liver tissues using nuclear and cytoplasmic extraction reagents.

### Western Blot Analysis

Western blot analysis was used to evaluated the effects of DOP on the expression of each protein in the whole cell, tissue, cytosol or nucleus. Equal amounts of protein (20 μg) were separated by 10∼15% sodium dodecyl sulfate polyacrylamide electrophoresis (10∼15% SDS-PAGE) and transferred to a nitrocellulose (NC) filter membrane. Then block the membrane with 5% non-fat milk in 0.13% Tween 80-1X TBS for 2∼3 h. Subsequently, the membrane incubated with the following specific antibodies anti-occludin, anti-ZO-1, anti-claudin-1, anti-Bax, anti-Bcl-2, anti-Caspase-3, anti-NF-κB, anti-IκBα, anti-p-IκBα, anti-α-SMA, anti-β-actin, anti-GAPDH and anti-Histone H3 overnight at 4°C. The membranes were then incubated with the anti-rabbit IgG or anti-mouse IgG secondary antibodies for 1 h. After treatment with an enhanced chemiluminescence method using the ECL kit, visualized with an Automated Imaging System (Gene Gnome5, Synoptics Ltd., United Kingdom). The protein band intensity was quantified with ImageJ software using β-actin, GAPDH or Histone H3 as a reference.

### Apoptosis Determination by Annexin V-FITC/PI Assay

Caco-2 cells were planted in plates and treated with LPS together with DOP at the same concentration as in the western blot experiments. After the induction of apoptosis using LPS and DOP, we used the trypsinization to harvest cells, wash twice in PBS, centrifuge at 1300 rpm for 3 min and resuspend in 500 μL of binding buffer. The DNA content was measured by flow cytometry after mixing the buffer with 5 μL of annexin V-FITC and adding 5 μL of propidium iodide (PI) for 5–10 min in the dark. Within 30 min after staining, at least 1 × 10^5^ cells/mL were collected by flow cytometer (Becton Dickinson, CA, United States) (Ghosh et al., 2011).

### Statistical Analysis

All results are expressed as the mean ± SD or ± SEM of more than three replicates for each prepared sample. GraphPad Prism version 5 was used for the statistical analysis. One-way analysis of variance (ANOVA) was used to evaluate the differences among groups.

## Results

### Chemical Structure of DOP

The phenol-sulfuric acid method determined the sugar content as 99.27%, suggesting that the purity of DOP was 99.27%. HPGPC chromatograms and the UV spectrum of DOP can be found in Supplementary Figure S1. The molecular weight of DOP was 1.95 (×105 Da. DOP showed no absorption at 260 nm or 280 nm in the UV spectra, indicating the absence of nucleic acids and proteins in DOP. The structure of DOP was elucidated in our previous study (Liu et al., 2020).

### DOP Alleviated Liver Fibrosis in a CCl_4_-Induced Hepatic Fibrosis Rat Model

**FIGURE 1:**
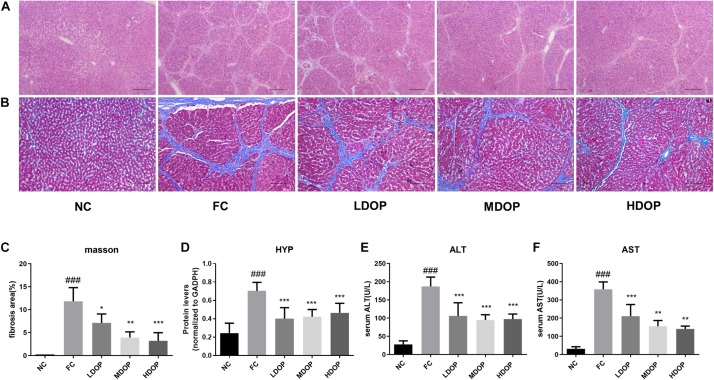
DOP alleviated hepatic fibrosis in a CCl_4_-induced liver fibrosis rat model. **(A)** Histological examination of liver slices with H&E staining (scar bar: 200 μm). **(B)** Histological examination of liver slices with Masson Trichrome staining (scar bar: 200 μm). **(C)** The area of fibrosis (% of the field). **(D)** The HYP contents in the liver homogenates. Serum ALT **(E)** and AST **(F)** levels. Data are presented as the mean ± S.D. (*n* ≥ 8). ^###^*p* < 0.001 vs. NC. *p < 0.05, **p < 0.01, and ***p < 0.001 vs. FC.

From the H&E stained sections of the liver (Figure 1A), hepatocytes in the normal group were observed to be arranged neatly with clear hepatic lobule structures and normal hepatic cell morphologies. The structure of the hepatic tissue in the model group was destroyed and replaced by a large number of proliferated fibrous tissues. Compared with the model group, the proliferation of fibrous tissues in the DOP-treated groups were reduced. Image-Pro Plus was used to calculate the degree of liver fibrosis in the Masson-stained images. Eight visual fields were selected from each slice to calculate the area of hepatic fibrosis. Only the NC group showed slight signs of fibrosis, whereas the model group showed large areas of fibrosis as well as the highest proportion of fibrosis. In contrast, the degree of fibrosis in the different concentration’s groups decreased after DOP administration, which illustrated that DOP could alleviate liver fibrosis in the CCl_4_-induced hepatic fibrosis model (Figures 1B,C). The HYP content of the NC group was lower than that of the model group, and the HYP content of the model group was the highest. After DOP (200, 400, and 800 mg/kg) were administered, the HYP contents in the liver decreased to 57.0 ± 6.0%, 60.3 ± 3.9%, and 63.2 ± 5.2% (Figure 1D), respectively, indicating that DOP could reduce collagen contents and reduce liver fibrosis.

### DOP Protected Liver Function in a CCl_4_-Induced Hepatic Fibrosis Rat Model

As shown in Figures 1E,F, compared with the normal group, ALT and AST levels in the model group increased by 6.73 ± 0.91-fold and 40.18 ± 0.11-fold, respectively. Moreover, ALT levels decreased to 56.59 ± 9.71%, 50.60 ± 3.88%, and 52.04 ± 3.66% and AST levels decreased to 58.71 ± 8.94%, 43.45 ± 9.97%, 39.25 ± 5.52% after DOP (200, 400, and 800 mg/kg) administration, respectively, indicating that DOP could improve liver function.

### DOP Reduced Intestinal Epithelial Cell Apoptosis in a CCl_4_-Induced Liver Fibrosis Rat Model

**FIGURE 2:**
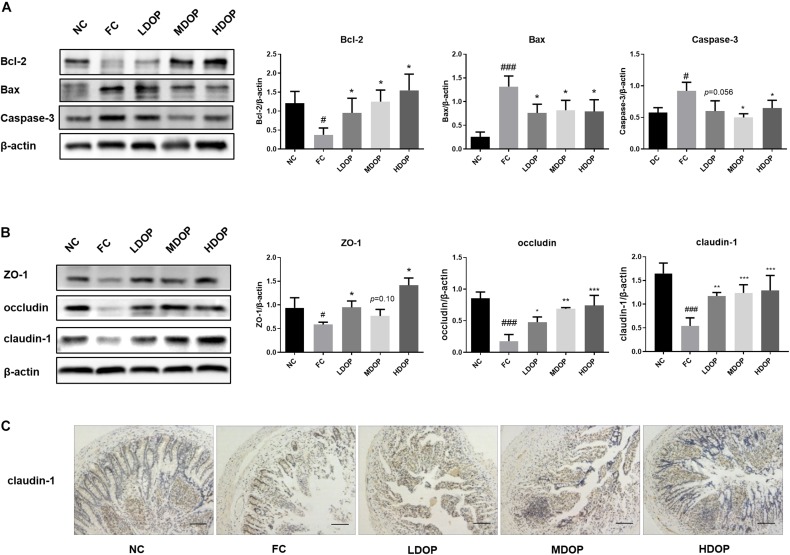
DOP protected the intestinal mucosal barrier and maintained intestinal epithelial integrity in the CCl_4_-induced liver fibrosis rat model. **(A)** The protein expression levels of Bcl-2, Bax and caspase-3 in the intestine. **(B)** The protein expression levels of ZO-1, occludin and claudin-1 in the intestine. **(C)** An immunohistochemical method was used to detect claudin-1. The images represented 2–3 different rats in each group (scar bar: 200 μm). Data are presented as the mean ± S.D. (n ≥ 3). ^#^*p* < 0.05 and ^###^*p* < 0.001 vs. NC. *p < 0.05, **p < 0.05, and ***p < 0.001 vs. FC. *p* = 0.10 vs. FC, and *p* = 0.056 vs. FC.

The structural basis and core content of the intestinal mucosal barrier is a mechanical barrier formed by the close connection between intestinal epithelial cells. To assess the impact of DOP on the intestinal tract in the CCl_4_ model, we first explored intestinal epithelial cell apoptosis. The western blot results suggested that compared with the normal group, the Bcl-2 expression was decreased by 3.67 ± 0.15-fold in the model group, but DOP (200, 400, and 800 mg/kg) significantly upregulated Bcl-2 protein levels (2.23 ± 0.42, 3.48 ± 0.29, 3.93 ± 0.40-fold), Moreover, the Bax and caspase-3 contents were the highest (5.92 ± 0.22, and 1.59 ± 0.10-fold compared higher than the normal group, respectively) in the model group. DOP diminished Bax and caspase-3 protein expression by approximately one-fold (Figure 2A). These results showed that the apoptotic rate of intestinal epithelial cells raised and the intestinal barrier was impaired in the model group. After administration of DOP, the apoptotic rate was reduced.

### DOP Enhanced Intestinal Mucosal Tight Junctions in a CCl_4_-Induced Liver Fibrosis Rat Model

ZO-1, occludin and claudin-1 are important tightly linked proteins. Decreased expression of these proteins might lead to increased cell permeability. DOP treatment led to an apparent restoration in ZO-1 protein expression, but we did not notice a significant difference in middle dose DOP treatment group (*p* = 0.01). Analogously, DOP could upregulate the expression of occludin by approximately three-fold and increase the expression of claudin-1 (Figure 2B).

The results from intestinal claudin-1 immunohistochemistry showed that the intestine of normal group was relatively complete, with a large number of cells and brown areas. In the model group, intestinal villi were seriously damaged, and almost no expression of claudin-1 was seen. After DOP administration, the villus structure in the intestine gradually recovered, and the brown areas were larger than those in the model group. Furthermore, claudin-1 expression was increased (Figure 2C). This illustrated that the intestinal mucosal epithelial barrier was damaged in the model group. The decrease in tight junctions between the cells increased the permeability between cells. The recovery of tight junctions between cells after DOP administration reduced the entry of toxic substances into the liver and alleviated liver fibrosis.

### DOP Restored the LPS-Mediated Caco-2 Cell Barrier and Reduced Intestinal Mucosal Cell Permeability

**FIGURE 3:**
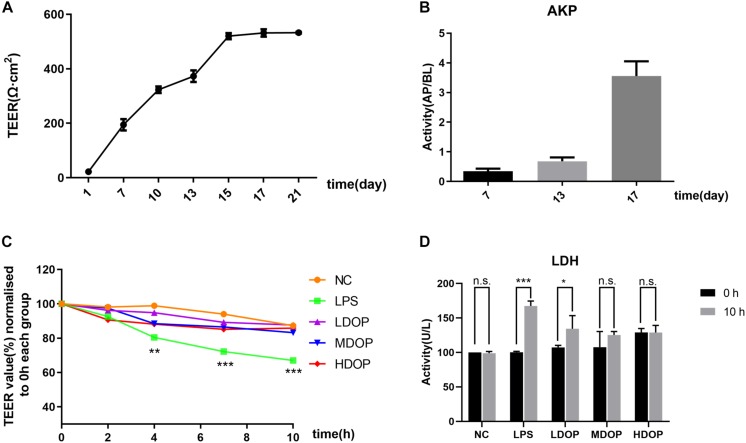
DOP restored the LPS-mediated Caco-2 cell barrier and reduced the permeability of intestinal mucosal cells. **(A)** The change in the TEER in the Caco-2 monolayer cell model over 21 days. **(B)** The AKP (AP/BL) activity ratio in the Caco-2 monolayer cell model. **(C)** The change in TEER after DOP administration. **(D)** The change in LDH contents before and after DOP administration. Data are presented as the mean ± S.D. (*n* ≥ 3). *p < 0.05, **p < 0.05, and ***p < 0.001 vs. FC, n.s., not significant.

We examined the activity of Caco-2 cells after administration of DOP by MTT assay. the result showed that DOP had no inhibitory effect on the activity of Caco-2 cells in the range of 0–400 μg/ml (Supplementary Figure S2). To evaluate the degree of damage to the cell membrane after LPS intervention and the alleviatory effects of DOP, a single layer Caco-2 epithelial cell model was established. Using a Millicell-ERS2 to measure resistance, as shown in Figure 3A, the resistance increased with time and began to grow slowly at day 15. It could be considered that the resistance value of the single layer model had reached the platform stage. Additionally, as shown in Figure 3B, the AKP contents of apical (AP) side were less than the basolateral (BL) side on the 7th day, while the AKP contents of the BL side were more than 10 times higher than the AP side on the 17th day. This proved the asymmetric distribution of the AKP and the obvious polarization phenomenon, which further demonstrated the establishment of the monolayer epithelial model. After LPS and DOP were given, the resistance values measured at different time points are shown in Figure 3C. The resistance values from the LPS group decreased by 30% at 10 h, while the resistance values from the DOP group were relatively the same to these of the NC group (Figure 3C). The LDH results showed that LDH leakage increased after LPS intervention, though LDH leakage in the DOP groups and NC group was relatively small (Figure 3D). It could be concluded that LPS destroyed the intestinal mucosal barrier and increased the intestinal cell permeability, while DOP repaired the intestinal mucosal barrier function and reduced the permeability between cells.

### DOP Decreased Apoptosis of Caco-2 Cells Mediated by LPS

**FIGURE 4:**
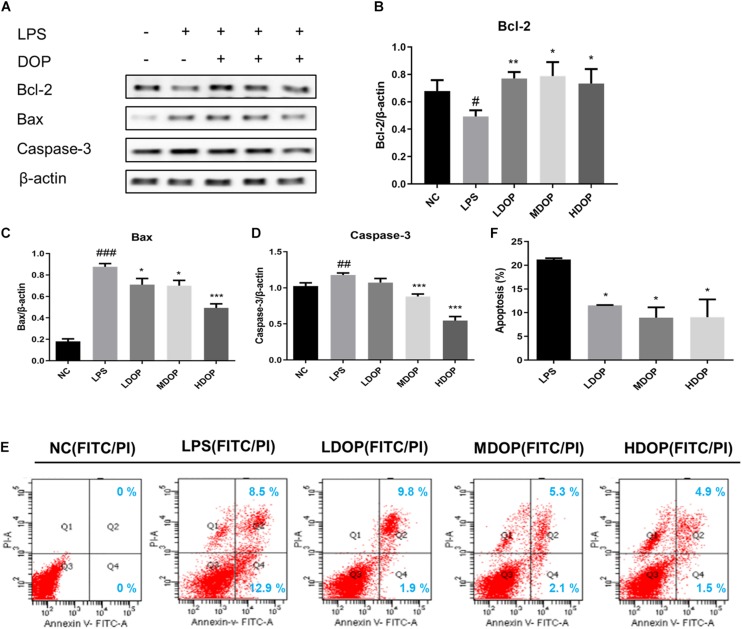
DOP decreased Caco-2 cell apoptosis mediated by LPS. **(A–D)** The protein expression levels of Bcl-2, Bax, and caspase-3 in Caco-2 cells. **(E)** Contour diagram of the FITC-AnnexinV/PI flow cytometry of Caco-2 cells. **(F)** Calculation of apoptotic cells according to **(D)** Data are presented as the mean ± S.D. (*n* ≥ 3). ^#^*p* < 0.05, ^##^*p* < 0.01, and ^###^*p* < 0.001 vs. NC. *p < 0.05, **p < 0.05, and ***p < 0.001 vs. FC.

To further confirm the effects of DOP on the apoptotic *in vitro*, we detected the expression of Bax, Bcl-2, and caspase-3 in LPS-induced Caco-2 cells by western blot. As shown in Figures 4A–D. DOP could upregulate the expression of Bcl-2 (approximately 1.5-fold) and downregulate the expression of Bax and caspase-3 in LPS-induced Caco-2 cells, which was consistent with the experimental results *in vivo*.

Annexin V FITC/PI staining was also applied to identify apoptotic patterns. Compared with the normal control group, the percentage of total apoptotic cells (early and late) treated with LPS and DOP is shown in Figure 4E and is counted in Figure 4F using a histogram. The results showed that the apoptotic rate decreased by 50% after DOP treatment, which were consistent with the western blot results. The results suggested that DOP could protect the intestinal mucosal barrier by inhibiting apoptosis.

### DOP Enhanced LPS-Mediated Tight Junctions in Caco-2 Cells

**FIGURE 5:**
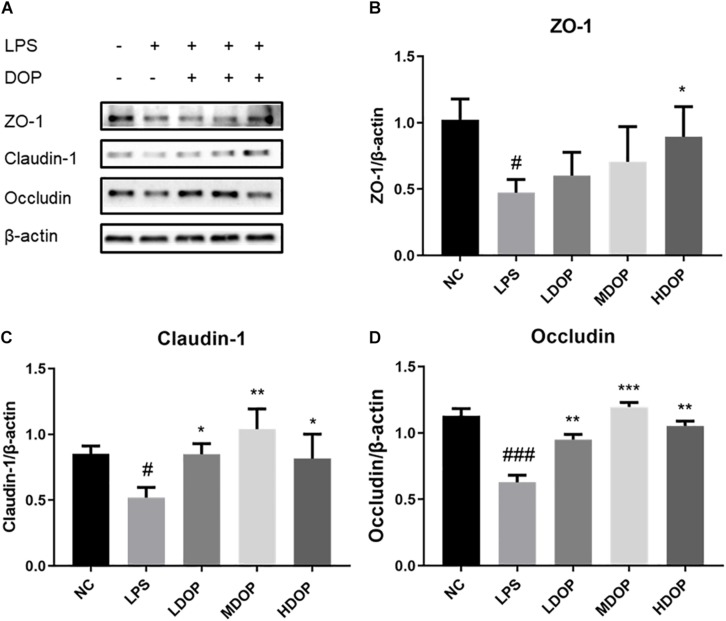
DOP enhanced the LPS-mediated tight junctions in Caco-2 cells mediated by LPS. **(A–D)** The protein expression levels of ZO-1, claudin-1 and occludin in Caco-2 cells. Data were presented as the mean ± S.D. (*n* ≥ 3). ^#^*p* < 0.05, and ^###^*p* < 0.001 vs. NC. *p < 0.05, **p < 0.05, and ***p < 0.001 vs. FC.

To examine whether DOP could protect the LPS-induced intestinal barrier function of Caco-2 cells, western blot was used to detect the expression of tight junction proteins in LPS-induced Caco-2 cells. As shown in Figure 5A, LPS caused apparent damage to Caco-2 cells, ZO-1, occludin, and claudin-1 protein levels being increased to varying degrees in DOP-treated cells (Figures 5B–D). This showed that DOP could protect the intestinal mucosal barrier by enhancing tight junction between cells.

### DOP Mitigated Liver Fibrosis Through the LPS-TLR4-NF-κB Pathway in a CCl_4_-Induced Liver Fibrosis Rat Model

**FIGURE 6:**
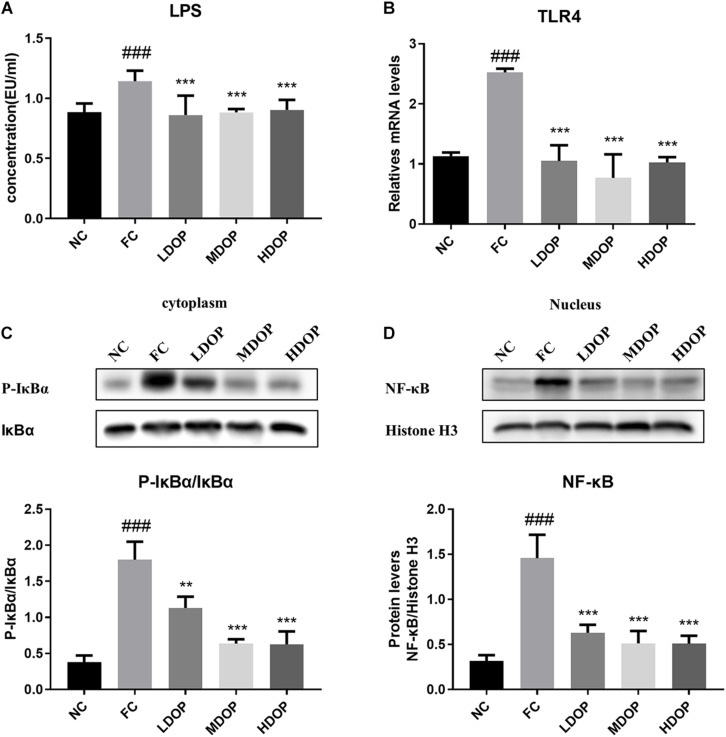
DOP mitigated liver fibrosis through the LPS-TLR4-NF-κB pathway in the CCl_4_-induced liver fibrosis rat model. **(A)** Serum LPS levels. **(B)** TLR4 mRNA levels. p-IκBα and IκBα protein levels **(C)** in the liver cytoplasm and NF-κB protein levels **(D)** in the liver nucleus. Data are presented as the mean ± S.D. (*n* ≥ 8). ^###^*p* < 0.001 vs. NC. **p < 0.05, and ***p < 0.001 vs. FC.

Although LPS in the intestinal cavity does not normally penetrate into the healthy intestinal epithelium, LPS may pass through the intestinal mucosa when the intestinal permeability is disturbed (Stevens et al., 2018). As shown in Figure 6A, compared with the normal group, LPS levels in the CCl_4_-induced rats increased by 40%, while after DOP treatment, LPS levels decreased by 20–25%, which indicates that DOP could restore intestinal mucosal injury. LPS can induce the activation of TLR4. We measured TLR4 mRNA level in the liver (Figure 6B) and found that TLR4 levels in the model group were significantly increased (3-fold), indicating that the TLR4 receptor was activated, Moreover, the TLR4 levels decreased by 1–3 times after DOP administration, indicating that activated TLR4 levels decrease, and the LPS contents entering through the intestinal mucosa to the liver also decrease. We next evaluated the nuclear translocation of NF-κB by western blot, and detected p-IκBα and IκBα protein levels in the cytoplasm. NF-κB was activated in the model group (4.6 times of the normal control group), and the level of p-IκBα/IκBα was also significantly increased (5-fold compared with the normal control group) (Figures 6C,D). DOP administration group significantly decreased NF-κB and p-IκBα content. It illustrated that DOP decreased NF-κB nuclear translocation.

### DOP Attenuated α-SMA and Collagen I by Modulating Inflammatory Cytokines in a CCl_4_-Induced Liver Fibrosis Rat Model

**FIGURE 7:**
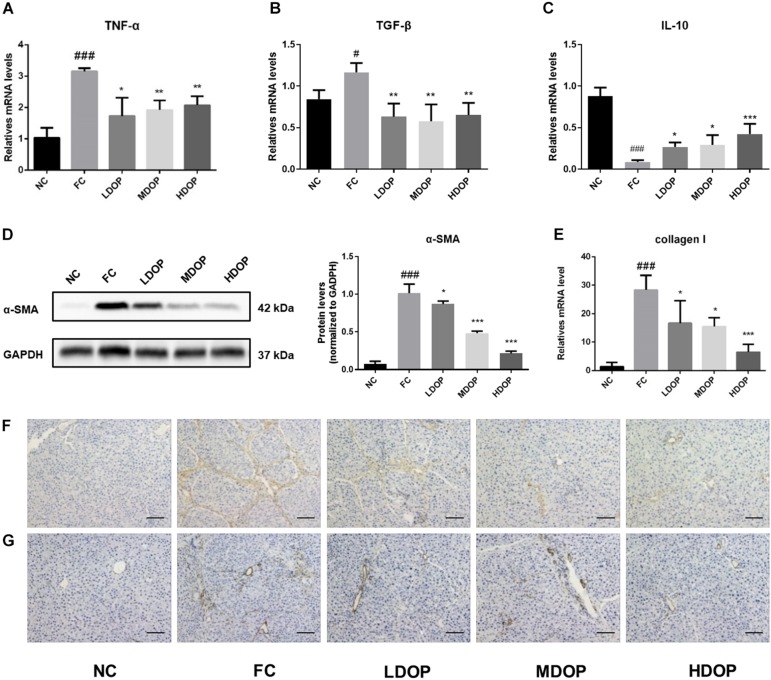
DOP attenuated α-SMA and collagen I by modulating inflammatory cytokines in the CCl_4_-induced liver fibrosis rat model. **(A–C)** The mRNA levels of TNF-α, TGF-β, IL-10 in the liver. **(D)** The protein expression levels of α-SMA in the liver. **(E)** The mRNA levels of collagen I in the liver. **(F–G)** An immunohistochemical method was used to detect α-SMA **(F)** and collagen I **(G)**. The images represented 2–3 different rats in each group (scale bar: 200 μm). Data are presented as the mean ± S.D. (*n* ≥ 3). ^#^*p* < 0.05, ^###^*p* < 0.001 vs. NC. *p < 0.05, **p < 0.05, and ***p < 0.001 vs. FC.

The levels of the pro-inflammatory cytokines TGF-β (Figure 7A) and TNF-α (Figure 7B) in the liver from the model group increased significantly while DOP treatment decreased the expression by approximately 30–40%. Oppositely, the levels of anti-inflammatory factor IL-10 (Figure 7C) were the lowest and the expression of inflammatory factors were out of control. After DOP administration, the expression of inflammatory factors gradually returned to normal level. A series of methods were used to analyze the expression of α-SMA and collagen I in hepatic fibrosis rats. Western experiments were used to analysis the protein contents of α-SMA in liver tissue. The expression of α-SMA in the CCl_4_-induced rats group increased significantly (15-fold compared with the normal control group) and decreased after DOP administration (Figure 7D). The expression of collagen I (Figure 7E) in liver tissue was measured by PCR, and the results were similar to those for α-SMA. Immunohistochemical experiments were carried out to further explore the expression of α-SMA and collagen I during the browning process and determine their localization in liver tissue (Figures 7F,G). The staining for α-SMA and collagen I were significantly increased, the degree of staining decreased after DOP administration. These results showed that DOP alleviated CCl_4_-induced liver fibrosis.

## Discussion

**FIGURE 8:**
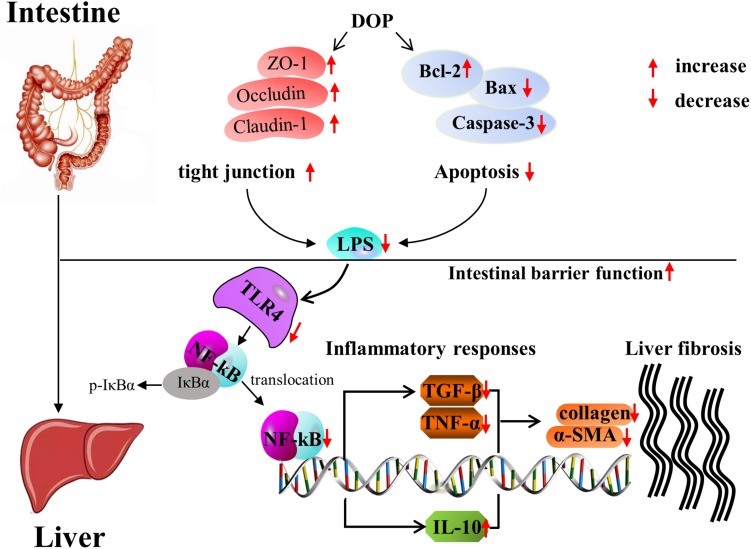
The mechanism of DOP treatment in CCl_4_-induced liver fibrosis through intestinal homeostasis and LPS-TLR4-NF-κB signaling.

Current research found that DOP could relieve liver fibrosis caused by CCl_4_ through the gut-liver axis. Further study of the mechanism indicated that DOP enhanced the tight junctions between intestinal cells and reduced intestinal cell apoptosis to maintain intestinal homeostasis. DOP reduced LPS entry into the liver to activate the TLR4/NF-κB signaling pathway, thereby reducing the production of inflammatory factors such as TGF-β and TNF-α, increasing the production of anti-inflammatory factors such as IL-10, and reducing the deposition of collagen to treat liver fibrosis (Figure 8).

Liver fibrosis is the early stage of liver cirrhosis but large amounts of evidence have suggested that fibrosis is reversible (Altrock et al., 2015). Liver fibrosis can occur through exposure to toxic substances such as acetaminophen, thioacetamide, chloroform and CCl_4_. CCl_4_ is considered a direct hepatotoxin that produces centrilobular necrosis and steatosis in the liver (Ma et al., 2014). In the current research, we discovered that DOP could reverse CCl_4_-induced inflammatory cell infiltration in rat hepatocytes and restore the normal hepatocytes morphology. These results showed the reversibility of liver fibrosis. Liver fibrosis is the consequence of chronic liver damage and extracellular matrix protein accumulation, such as α-SMA and Collagen I (Bataller and Brenner, 2005). Hepatic mRNA expression of Collagen I was increased in CCl_4_-induced rats and significantly inhibited after DOP treatment. Western blot experiments established that DOP decreased the levels of α-SMA in CCl_4_-induced rats. It was in accordance with the immunohistochemical analysis. These results indicated that DOP had inhibitory effect on the formation of liver fibrosis caused by CCl_4_.

It has been reported that, alcohol can change the number of intestinal bacteria and inhibit intestinal movement in alcoholic liver disease, leading to overgrowth of Gram-negative bacteria. LPS levels increased in portal vein blood and increased TNF-α production through TLR4/NF-κB (Szabo, 2015). TNF-α induces neutrophil infiltration, stimulates the production of mitochondrial oxidants, and activates and stimulates inflammation and fibrosis signals (Bataller and Brenner, 2005). We wanted to know whether CCl_4_-induced liver fibrosis is relevant to the intestinal tract. The gastrointestinal epithelium is a multilayer barrier that plays a role in the defense of intestinal pathogens and other harmful compounds in the intestinal tract (Deng et al., 2018). The epithelial barrier comprises epithelial cells and transmembrane proteins of TJs, namely, ZO-1, occludin and claudin-1. TJs play a significant role in the formation and maintenance of intestinal epithelial barrier integrity (Lerner and Matthias, 2015). Destruction of TJ-related barrier integrity leads to increased intestinal permeability and increased inflammation (Wen et al., 2014). Previous studies have demonstrated that “Xia-Yu-Xue Decoction” reversed histological changes caused by CCl_4_ and restored intestinal barrier integrity (Ma et al., 2017). We found that the expression of occludin and claudin-1 in the rat intestinal tract decreased significantly in CCl_4_-induced liver fibrosis rats, while DOP enhanced the expression of these proteins. Besides, the LPS contents in the serum were reduced in the DOP treatment groups compared with these in the FC group. It was concluded that DOP enhanced the tight junctions between cells and inhibited the transfer of LPS to the liver. We also found that the decrease in tight junction proteins was accompanied by cell apoptosis. Intestinal cell apoptosis was higher in FC group than in the DOP-treated groups. This suggested that the intestinal mucosal barrier might be maintained by tight junctions and apoptotic proteins. DOP protected the intestinal mucosal barrier by strengthening the tight junctions between cells and alleviating apoptosis.

Transformed cancer-derived intestinal cell lines have been used to model the human intestine due to their ability to replicate intestinal permeability and membrane transport and include T84, Caco-2, and HT29 cells (Yu et al., 2017). Caco-2 cells are the most generally used *in vitro* model to study the structure and function of the intestinal tract (Tu et al., 2016). Therefore, we further investigated the effects of DOP on LPS-treated Caco-2 cells. AKP activity and LDH leakage are two differentiation markers (Aziz et al., 2014). After 21 days of cell culture, the enhancement of these two factors confirmed the differentiation of Caco-2 cells. *Shen, Li et al*. have illustrated that LPS caused a sharp decline in TEER and downregulated the expression of tight junction proteins (Chen et al., 2018; Li et al., 2018). Current research found that DOP can relieved the reduce of TEER induced by LPS and upregulate the level of tight junction protein induced by LPS. We also found that DOP could reduce LPS-treated Caco-2 cell apoptosis, suggesting that DOP might protect the intestinal mucosal barrier and maintain intestinal homeostasis. However, there were still some limitations in our article. The main focus of our work was to study how DOP regulates liver fibrosis through the intestinal tract; thus, cell experiments will continue in future research.

TLR4 has been reported to be the main sensor for detecting various bacterial activities, especially bacterial toxins, and to activate natural immune responses (Kawai and Akira, 2007). NF-κB is a vital nuclear transcription factor and is considered essential for the control of pro-inflammatory genes. It is regarded as an important target for the treatment of inflammatory diseases (Oberg et al., 2009; Pei et al., 2010). It has been reported that the TLR4/NF-κB signaling pathway is an important mechanism for regulating immune and inflammatory responses (Peng et al., 2014). When LPS binds to TLR4, it can activate IκBα kinase, which can phosphorylate IκBα, and release NF-kB from the cytoplasmic NF-kB/IκBα complex, subsequently activating and exposing the nucleus (Yuan et al., 2015). Our observations in this study suggested that DOP treatment inhibited TLR4 activation and blocked TLR4 signal transduction to promote NF-κB activation. These results confirmed that DOP could alleviate toxicity and inflammation induced by CCl_4_ through the TLR4/NF-κB signal pathway. TNF-α and TGF-β are activated by NF-κB in CCl_4_-induced rats (Tsukada et al., 2006; Yu et al., 2007). Therefore, CCl_4_, as a key and determinant factor in the pathological development of liver fibrosis induced by TLR4/NF-κB activation, was used to clarify whether DOP had potential anti-inflammatory and oxidative stress effects and determine the possible mechanism between them. We found that DOP remarkable restrain the delivery of inflammatory genes. These findings indicated that DOP treatment reduced CCl_4_-induced inflammation, suggesting that DOP might alleviate liver fibrosis by inhibiting inflammation through the TLR4/NF-κB pathway.

## Conclusion

In conclusion, DOP is a potential adjuvant therapy for the treatment of liver fibrosis. DOP could protect the intestinal mucosal barrier, reduce intestinal cell permeability and maintain intestinal homeostasis by enhancing tight junctions in intestinal cells and alleviating apoptosis. In contrast, DOP could inhibit the activation of the TLR4/NF-κB signaling pathway, thereby reducing the production of inflammatory factors such as TGF-β and TNF-α, and increasing the production of anti-inflammatory factors such as IL-10, therefore playing a significant role in preventing liver fibrosis. Therefore, this study indicates that the protective effects of DOP on hepatic fibrosis might be achieved by protecting the intestinal mucosal barrier and regulating the TLR4/NF-κB pathway. If proper drug interventions can target regulatory mechanism of intestinal homeostasis, they may provide a new strategy for the prevention and treatment of liver fibrosis.

## Data Availability Statement

All datasets generated for this study are included in the article/Supplementary Material.

## Ethics Statement

The animal study was reviewed and approved by the Institutional Animal Care and Use Committee of Tongji Medical College, Huazhong University of Science and Technology.

## Author Contributions

KW, XY, and ZW carried out experiments. RY and YZ participated in experiment design. XY, QL, and HW analyzed data and wrote the manuscript. XY and HM contributed to the revision of manuscript.

## Conflict of Interest

The authors declare that the research was conducted in the absence of any commercial or financial relationships that could be construed as a potential conflict of interest.

## Abbreviations

α-SMA: α-smooth muscle actin
AKP: alkaline phosphatase
ALT: alanine aminotransferase
AST: aspartate aminotransferase
CCl_4_: carbon tetrachloride
DOP: *Dendrobium officinale* polysaccharide
ECM: extracellular matrix
FC: fibrotic control
HDOP: high DOP treated group
HSCs: hepatic stellate cells
HYP: hydroxyproline
IL-10: interleukin-10
LDH: lactate dehydrogenase
LDOP: low DOP treated group
LPS: lipopolysaccharides
MDOP: middle DOP treated group
NC: normal control
NF- κ B: nuclear factor- κ B
TGF- β: transforming growth factor-beta
TJ: tight junction
TLR4: toll-like receptor 4
TNF- α: tumor necrosis factor-alpha.
