# Supplementary material for: Dendrobium officinale Polysaccharide Protected CCl4-Induced Liver Fibrosis Through Intestinal Homeostasis and the LPS-TLR4-NF-κB Signaling Pathway
Source: Front Pharmacol. 2020 Mar 12;11:240. doi: 10.3389/fphar.2020.00240 (PMC7080991; doi:10.3389/fphar.2020.00240)
Supplement: Supplementary file 1 [file Data_Sheet_1.DOCX]

**Supplementary method**

1. *phenol-sulfuric acid method*

The phenol-sulfuric acid method in this study is as follows (Zhang et al., 2020): (1) 1 mL of DOP aqueous solution was mixed with 0.6 mL of 5 % aqueous solution of redistilled phenol in a test tube. (2) 3 mL of concentrated sulfuric acid was added to the test tube with the mixture, then the test tube was placed in boiling water bath for 20 min. (3) The test tube placed at room temperature for 15 min. (4) The light absorption at 490 nm was measured on the spectrophotometer and the carbohydrate concentration was determined according to a standard curve.

1. *high performance gel permeation chromatography (HPGPC)*

The molecular weight of DOP was determined by high performance gel permeation chromatography (HPGPC) on an Agilent-LC 1100 instrument (Agilent, USA) fitted with TSK-gel G4000PWxl (7.8 mm × 300 mm) column and the column temperature was kept at 30 ℃. In brief, the purified polysaccharide was dissolved in the mobile phase (0.05 M Na_2_SO_4_) and passed through a 0.22 μm filter. Then the sample solution (1 mg/mL) was eluted with 0.05 M Na_2_SO_4_ solution at a flow rate of 1.0 mL/min and detected by a SHIMADZU refractive index detector (RAD-10A). The column was calibrated with T-series dextrans (T-5, 10, 40, 70, 500, 2000 kDa) (Sigma, USA)(Liu et al., 2019).

1. *UV-spectrophotometer*

The UV spectra of the samples (2 mg mL^−1^) were recorded in the region of 200–400cm^−1^ for protein and nucleic acid detection with a UV spectrophotometer (model UV-1750).

1. *monosaccharide composition analysis*

The monosaccharide composition was measured by HPLC using the procedure described in our previous report (Zhang et al., 2019). DOP was hydrolyzed into monosaccharides with 4 M trifluoroacetic acid (TFA) at 100 ℃ for 4 h. The hydrolyzed sample was derivatized with 1-phenyl-3-methyl-5-pyrazolone (PMP) and analyzed by a HPLC system (Agilent, USA). The monosaccharide standards, D-mannose, L-rhamnose, D-glucuronic acid, D-galacturonic acid, D-glucose, D-galactose, D-xylose and L-arabinose, were also derivatized with PMP using the same method as for the derivatization of DOP. After derivatization, the samples were analyzed on a ZORBAX Eclipse XDB-C18 HPLC column (4.6 mm × 50 mm, Agilent, USA) at 30 °C. The mobile phase consisted of phosphate buffer (0.1 M, pH 6.7) and acetonitrile in a ratio of 83:17 (v/v, %). The wavelength for UV detection was 245 nm.

**References:**

Zhang, W., Wu, J., Weng, L., Zhang, H., Zhang, J., and Wu, A. (2020). An improved phenol-sulfuric acid method for the determination of carbohydrates in the presence of persulfate. Carbohyd. Polym. 227, 115332. doi: 10.1016/j.carbpol.2019.115332.

Liu, Y., Yang, L., Zhang, Y., Liu, X., Wu, Z., Gilbert, R.G., et al. (2019). Dendrobium officinale polysaccharide ameliorates diabetic hepatic glucose metabolism via glucagon-mediated signaling pathways and modifying liver-glycogen structure. Journal of Ethnopharmacology, 112308. doi: <https://doi.org/10.1016/j.jep.2019.112308>.

Zhang, Y., Pan, X., Ran, S., and Wang, K. (2019). Purification, structural elucidation and anti-inflammatory activity in vitro of polysaccharides from Smilax china L. Int J Biol Macromol 139, 233-243. doi: 10.1016/j.ijbiomac.2019.07.209.
