# Supplementary material for: Dendrobium officinale Polysaccharide Protected CCl4-Induced Liver Fibrosis Through Intestinal Homeostasis and the LPS-TLR4-NF-κB Signaling Pathway
Source: Front Pharmacol. 2020 Mar 12;11:240. doi: 10.3389/fphar.2020.00240 (PMC7080991; doi:10.3389/fphar.2020.00240)
Supplement: Supplementary file 2 [file Data_Sheet_2.docx]

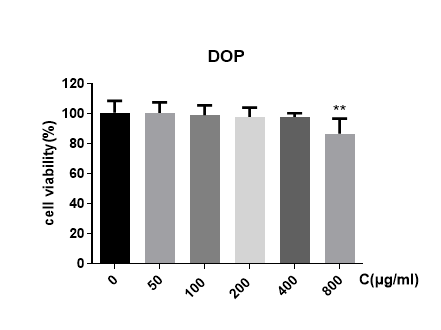

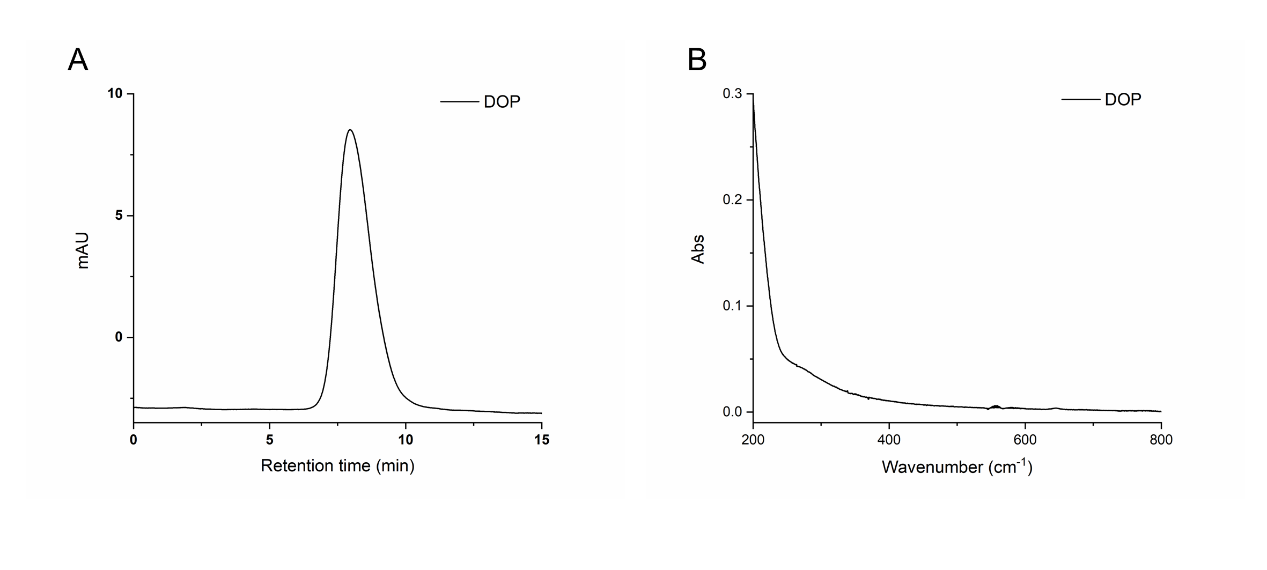
Figure S1. (A) HPGPC chromatogram of DOP, (B) UV spectrum of DOP.

Figure S2. Effects of DOP at different concentrations on Caco-2 cells. (DOP concentration range is 0-800μg / ml)
